# Molecularly defined unfolded protein response subclasses have distinct correlations with fatty liver disease in zebrafish

**DOI:** 10.1242/dmm.014472

**Published:** 2014-07

**Authors:** Ana M. Vacaru, Antonio Fabio Di Narzo, Deanna L. Howarth, Orkhontuya Tsedensodnom, Dru Imrie, Ayca Cinaroglu, Salma Amin, Ke Hao, Kirsten C. Sadler

**Affiliations:** 1 Department of Medicine/Division of Liver Diseases, Icahn School of Medicine at Mount Sinai, New York, NY 10029, USA.; 2 Department of Developmental and Regenerative Biology, Icahn School of Medicine at Mount Sinai, Box 1020, 1 Gustave L. Levy Place, New York, NY 10029, USA.; 3 Department of Genetics and Genomic Sciences and Icahn Institute for Genomics and Multiscale Biology, Icahn School of Medicine at Mount Sinai, New York, NY 10029, USA.; 4 Graduate School of Biomedical Sciences, Icahn School of Medicine at Mount Sinai, New York, NY 10029, USA.

**Keywords:** Unfolded protein response, Steatosis, Zebrafish, Tunicamycin, Thapsigargin, ER stress, Fatty liver disease

## Abstract

The unfolded protein response (UPR) is a complex network of sensors and target genes that ensure efficient folding of secretory proteins in the endoplasmic reticulum (ER). UPR activation is mediated by three main sensors, which regulate the expression of hundreds of targets. UPR activation can result in outcomes ranging from enhanced cellular function to cell dysfunction and cell death. How this pathway causes such different outcomes is unknown. Fatty liver disease (steatosis) is associated with markers of UPR activation and robust UPR induction can cause steatosis; however, in other cases, UPR activation can protect against this disease. By assessing the magnitude of activation of UPR sensors and target genes in the liver of zebrafish larvae exposed to three commonly used ER stressors (tunicamycin, thapsigargin and Brefeldin A), we have identified distinct combinations of UPR sensors and targets (i.e. subclasses) activated by each stressor. We found that only the UPR subclass characterized by maximal induction of UPR target genes, which we term a stressed-UPR, induced steatosis. Principal component analysis demonstrated a significant positive association between UPR target gene induction and steatosis. The same principal component analysis showed significant correlation with steatosis in samples from patients with fatty liver disease. We demonstrate that an adaptive UPR induced by a short exposure to thapsigargin prior to challenging with tunicamycin reduced both the induction of a stressed UPR and steatosis incidence. We conclude that a stressed UPR causes steatosis and an adaptive UPR prevents it, demonstrating that this pathway plays dichotomous roles in fatty liver disease.

## INTRODUCTION

The unfolded protein response (UPR) allows cells to adapt to changing physiological demands and to extreme stress by enhancing protein folding and quality control in the endoplasmic reticulum (ER) and by modulating the influx of nascent proteins into the ER. The responses to different stressors are nuanced and dynamic, eliciting different combinations of UPR sensor and target gene activation that fluctuate with the nature and duration of the stressor. Importantly, these different UPRs have different outcomes, which can alternatively prevent or promote disease.

UPR activation provides a first-line defense against secretory pathway stress by reducing the unfolded protein burden in the ER and restoring homeostasis. In this case, UPR activation is beneficial, as it allows cells to withstand and adapt to stress (Rutkowski and Kaufman, 2007). Chronic, robust UPR induction, however, can lead to cell death. ER stress-induced apoptosis is proposed to be central to several diseases (Wang and Kaufman, 2012), and much effort has been focused on defining aspects of the ‘terminal UPR’ that is induced in cells overtaxed by secretory demand (Papa, 2012; Tabas and Ron, 2011). Computational modeling of the response to unmitigated ER stress has identified characteristics of terminal and adaptive UPRs (Erguler et al., 2013), which we term subclasses (Imrie and Sadler, 2012). However, such models have not been applied to the understanding of UPR dynamics *in vivo*. Moreover, little is known about how different stressors translate to clinically relevant outcomes (Walter and Ron, 2011).

The UPR is comprised of interconnected pathways (Bernales et al., 2006; Walter and Ron, 2011) regulated by three activators or ‘sensors’. Activating transcription factor 6 (ATF6) regulates the main ER chaperone BiP, as well as other components of the protein folding machinery, and induces transcription of itself and of *XBP1* (Shoulders et al., 2013), a downstream target of a second UPR sensor, inositol-requiring enzyme-1a (IRE1A, ERN1). Unfolded proteins bind and activate IRE1A (Gardner and Walter, 2011) to splice *XBP1* mRNA to produce *XBP1s,* which encodes a transcription factor that collaborates with ATF6 to induce the UPR transcriptome (Shoulders et al., 2013). Protein kinase RNA-like endoplasmic reticulum kinase (PERK, EIF2AK3) is a kinase and the third UPR sensor. It phosphorylates EIF2A, promoting translation inhibition, which results in reduction of the secretory cargo load in the ER (Harding et al., 2000b), but also selectively inducing translation of ATF4 (Harding et al., 2000a), another transcription factor that activates a distinct set of UPR responsive genes.

Drugs that directly block key ER functions are commonly used to study the UPR and typically cause full activation of all three sensors and most UPR target genes. For instance, tunicamycin (Tm) blocks protein N-linked glycosylation and thapsigargin (Tg) disrupts ER calcium homeostasis, each creating a backlog of terminally unfoldable proteins. Transient or low-level exposure to these drugs induces a UPR that can resolve the unfolded protein load in the ER and, thus, secretory pathway function is maintained during low level or acute stress. However, exposure to drug levels that induce a cargo burden that the UPR cannot overcome results in secretory organelle dysfunction, impaired protein secretion and chronic UPR activation. This is ER stress; however, this term is frequently more broadly applied to cells with any measure of UPR activation. Such broad usage has generated confusion in the field, because it is based on the assumption that any type of UPR activation is equated with cellular stress and ER dysfunction. However, in many cases, the UPR becomes activated in response to secretory demands that are part of normal physiology and thus are not stress. Aside from the well-studied terminal UPR subclass, which leads to cell death (Papa, 2012; Pyati et al., 2011; Tabas and Ron, 2011), other UPR subclasses have not been defined *in vivo*.

**TRANSLATIONAL IMPACT**

**Clinical issue**

Defects in the protein secretory pathway trigger activation of the unfolded protein response (UPR). The term endoplasmic reticulum (ER) stress is frequently used to describe all scenarios where UPR activation is detected, leading to a simplistic model whereby UPR activation is equated with pathology. However, although some stimuli that cause a robust UPR can induce apoptosis and contribute to disease, low level or transient UPR activation restores cell function, maintains homeostasis and can adapt cells to withstand further stress. UPR activation is detected in many pathologies, including fatty liver disease, the most common hepatic pathology in the Western world. A central and unanswered question in the field is whether all ‘flavors’ (i.e. subclasses) of UPR activation generate this pathological outcome.

**Results**

In this study, the authors define three distinct UPR subclasses that are induced in the liver of zebrafish larvae exposed to three commonly used stressors, tunicamycin, thapsigargin and Brefeldin A. Each stressor induces a unique panel of UPR sensors and target genes, which indicates that the UPR response *in vivo* is highly nuanced. The most robust induction of UPR sensors and target genes, which the authors term a ‘stressed UPR’, is achieved by exposure to high tunicamycin concentrations over 48 hours and by the maximal tolerable dose of thapsigargin. Importantly, only this stressed UPR induces fatty liver disease. The authors report that principal component analysis of UPR target gene induction serves as a marker of the stressed UPR in zebrafish livers and that this same principal component applied to samples from patients with fatty liver disease reveals UPR induction in these individuals. Interestingly, the induction of an adaptive UPR by pretreatment of larvae with thapsigargin reduces the induction of a stressed UPR by tunicamycin and reduces steatosis.

**Implications and future directions**

These data demonstrate that, despite the widespread reports of ER stress-associated fatty liver disease, only a distinct UPR subclass can cause this disease. This finding suggests that the current usage of the term ‘ER stress’ should be revised to refer only to a stressed UPR that causes cell dysfunction. Notably, these findings also define an adaptive UPR that protects cells from subsequent stress and prevents fatty liver. Thus, the UPR plays a dichotomous role in fatty liver disease, which raises the possibility that induction of an adaptive UPR could be exploited therapeutically to reduce fatty liver disease.

Fatty liver disease (FLD) is characterized by lipid accumulation in hepatocytes (steatosis). Alcohol abuse and obesity are the most common causes of FLD, one of the most common hepatic pathologies in the Western world (Cohen et al., 2011). Markers of UPR activation have been detected in FLD samples from multiple species (Imrie and Sadler, 2012; Malhi and Kaufman, 2011) and inducing ER stress with Tm is sufficient to cause FLD in mice (Lee et al., 2012; Rutkowski et al., 2008; Teske et al., 2011; Wu et al., 2007; Yamamoto et al., 2010; Zhang et al., 2011) and zebrafish (Cinaroglu et al., 2011; Thakur et al., 2011). Moreover, steatosis can be influenced by altering the expression of key UPR mediators, including BiP (Ji et al., 2011; Kammoun et al., 2009; Ye et al., 2010), PERK (Teske et al., 2011), IRE1A/XBP1 (Lee et al., 2008; Ozcan et al., 2006; Zhang et al., 2011) and ATF6 (Cinaroglu et al., 2011; Howarth et al., 2014; Rutkowski et al., 2008; Wu et al., 2007; Yamamoto et al., 2010). Our recent finding that Atf6 overexpression is sufficient to cause steatosis in zebrafish (Howarth et al., 2014) definitively shows that activation of this pathway is a culprit in FLD. This study indicates that Atf6 causes steatosis by inducing lipid synthesis; however, there are probably other aspects of the UPR that contribute to FLD. Identifying these requires defining which aspects of the UPR are associated with steatosis.

Studies in yeast (Jonikas et al., 2009; Thibault et al., 2011) and mammalian cells *in vitro* (DuRose et al., 2006) reveal a range in the amplitude and kinetics of each UPR sensor. However, virtually nothing is known about the comparable response of vertebrate cells *in vivo*. We used zebrafish larvae to ask whether all stressors that induce unfolded protein accumulation in the ER are able to cause steatosis across a large population of animals. Our previous studies demonstrated that both ethanol (Howarth et al., 2011; Passeri et al., 2009; Tsedensodnom et al., 2013) and Tm (Cinaroglu et al., 2011; Howarth et al., 2013) cause a high incidence of steatosis in zebrafish larvae, but mRNA expression analysis of a few UPR target genes suggested that response to these stressors was distinct (Cinaroglu et al., 2011; Howarth et al., 2012; Tsedensodnom et al., 2013). Here, we systematically analyzed the UPR in response to different stressors by measuring multiple aspects of secretory pathway function and UPR activation. These parameters were then correlated with steatosis incidence. We identified distinct UPR subclasses and found that only those that caused maximal induction of a panel of UPR target genes (termed a stressed UPR) caused steatosis. In contrast, an adaptive UPR protected against steatosis. We conclude that not all UPR subclasses cause FLD and that inducing an adaptive UPR could be exploited therapeutically to reduce FLD.

## RESULTS

### Tm is the most efficient steatosis-inducing stressor

Tm causes UPR activation and steatosis in all species studied (Cinaroglu et al., 2011; Rutkowski et al., 2008; Teske et al., 2011; Thakur et al., 2011; Yamamoto et al., 2010; Zhang et al., 2011) but whether other stressors also cause FLD is not known. We treated zebrafish larvae with Tm and two other commonly used secretory pathway stressors, Tg and Brefeldin A (BFA), and assessed steatosis and UPR induction in the liver. All treatment protocols used larvae during developmental stages when hepatocytes become functional [i.e. 3 days post fertilization (dpf) or later] and prior to 6 dpf, when the yolk has been fully consumed and the larvae depend on external nutrients.

Tg blocks the calcium pump in the ER membrane and thus inhibits the function of the many chaperones that require high calcium levels. Tg concentrations of 1 μM or higher were lethal (supplementary material Fig. S1A), and thus 0.75 μM was chosen as the maximal tolerable dose for chronic exposure (48 hours, 3–5 dpf).

**Fig. 1.**
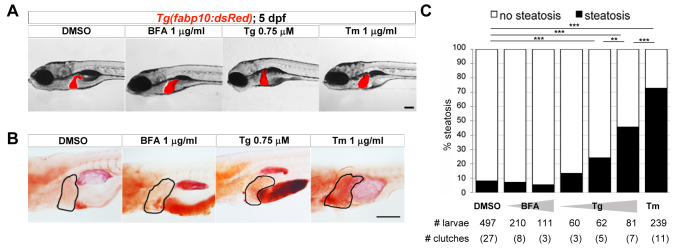
**Tm is the only ER stressor that induces fatty liver.** (A,B) Representative images of 5-dpf larvae treated for 48 hours with DMSO, BFA, Tg or Tm at the indicated concentrations. (A) Live transgenic *Tg(fabp10:dsRed)* larvae were imaged. (B) Fixed larvae were stained for neutral lipids with Oil Red O. Livers are circled. (C) Larvae treated with DMSO, 1 and 2 μg/ml BFA, 0.25, 0.5 and 0.75 μM Tg or 1 μg/ml Tm from 3–5 dpf were stained with Oil Red O and scored for the presence (black bars) or absence (white bars) of steatosis. The total number of larvae scored and the number of clutches are indicated. **P<0.01, ***P<0.0001, calculated using Fisher’s exact test. Scale bars: 200 μm (A), 1 mm (B).

BFA inhibits ADP-ribosylation factor 1 (Robineau et al., 2000) and thus blocks vesicle trafficking from the ER to the Golgi complex (GC), causing ER-GC fusion and secretory cargo accumulation. We chose 1 μg/ml as the optimal BFA dose because it was well tolerated (supplementary material Fig. S1B; Fig. 1A) and highly effective, as measured by complete GC fragmentation in hepatocytes (supplementary material Fig. S1C), enterocytes and chondrocytes (supplementary material Fig. S1D).

Tm inhibits DPAGT1 (Eckert et al., 1998; Lehrman et al., 1988), the enzyme required for linking dolichol to the oligosaccharide that serves as a precursor for N-linked glycosylation (Lehrman, 1991), causing hypoglycosylated proteins to accumulate in the ER. The maximal tolerable Tm dose for exposing 3–5 dpf larvae was 1.5 μg/ml (supplementary material Fig. S1E). Efficacy was determined using *Tg(−3.5fabp10a:gc-EGFP)*^lri500^ transgenic zebrafish, which express a secreted glycoprotein, vitamin D-binding protein (Gc), fused to enhanced green fluorescent protein (EGFP) under the hepatocyte-specific *fabp10* promoter (Xie et al., 2010). We assessed the glycosylation status of Gc-EGFP by immunoblotting with anti-GFP: the fully glycosylated form of Gc-EGFP migrated at 90 kDa and a hypoglycosylated, faster migrating moiety was detected in fish treated with Tm and in control lysates treated with peptide-*N*-glycosidase (PNGase) to remove all glycans (supplementary material Fig. S1F). Importantly, neither BFA nor Tg impaired Gc-EGFP glycosylation (supplementary material Fig. S1F) and none of the stressors significantly affected total Gc-EGFP expression (supplementary material Fig. S1F,G). These data illustrate both Tm efficacy and show that any effects caused by Tg or BFA treatment cannot be attributed to a secondary effect of blocking N-glycosylation, consistent with findings in mammalian cells (Sampath et al., 1992).

We analyzed larvae treated with each stressor from 3–5 dpf for gross morphology and liver size and shape using transgenic fish that express red fluorescent protein (RFP) in hepatocytes [*Tg(fabp10:RFP)*] (Fig. 1A) and steatosis incidence by Oil Red O staining (Fig. 1B,C). All stressors caused similar gross morphological defects, including slight liver enlargement, failure to inflate their swim bladder and moderate reduction in head and eye size, although larvae treated with 0.75 μg/ml Tg appeared more severely affected than those treated with Tm or BFA (Fig. 1A).

Tm at 1 μg/ml was the most efficient steatosis-inducing stressor, with strong Oil Red O staining observed in the liver of 73% of treated larvae (*n*=239 larvae in 11 clutches; *P*<0.0001), compared with 8% in DMSO-treated larvae (*n*=497 larvae from 27 clutches; Fig. 1C). In contrast, only 46% of fish treated with 0.75μM Tg (*n*=81 larvae from 7 clutches, *P*<0.0001) and less than 30% of larvae treated with lower Tg concentrations displayed hepatosteatosis (Fig. 1C). BFA treatments of 1 or 2 μg/ml showed a steatosis incidence indistinguishable from controls (Fig. 1C). These data demonstrate that different mechanisms of inducing secretory pathway stress have dramatically different outcomes in the liver.

### Different stressors induce distinct UPR subclasses in the liver

**Fig. 2.**
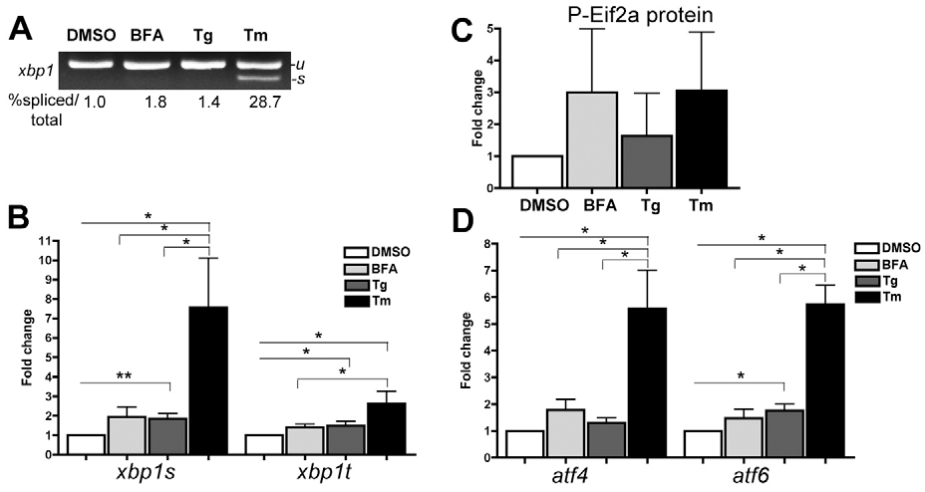
**BFA, Tg and Tm differentially induce UPR sensors in the liver.** (A,B) Standard PCR (A) and qPCR (B) analysis of *xbp1* splicing in the livers of fish treated from 3 to 5 dpf with DMSO, 1 μg/ml BFA, 0.75 μM Tg or 1 μg/ml Tm using primers that amplify both unspliced (-*u*) and spliced (-*s*) *xbp1*. The average ratio of *xbp1-second* to *xbp1-t* is indicated (*n*=2). (C) Protein extracts from livers dissected from 5-dpf larvae treated as in A were blotted with anti-*P-Eif2a* and anti-*tubulin* as a loading control. Quantification and normalization to tubulin and DMSO controls is shown (*n*=2). (D) Analysis of *atf4* and *atf6* expression in livers of fish treated as in A. In B and D, target gene expression was normalized to *rpp0* and fold changes compared to DMSO are plotted; *n*=13 for DMSO, 10 for Tm, 8 for Tg and 6 for BFA. Bars represent standard error in all graphs. *P<0.05, **P<0.01, calculated using paired Wilcoxon test.

We hypothesized that the different outcomes caused by BFA, Tg and Tm were attributed to variations in UPR activation. *xbp1* splicing as a surrogate for Ire1a/Ern1 activation was measured by standard PCR (Fig. 2A) and quantitative PCR (qPCR, Fig. 2B). Eif2a phosphorylation was used to assess Perk activation (Fig. 2C; supplementary material Fig. S2A); *atf4* and *atf6* expression served to assess the induction of these UPR mediators (Fig. 2D) because antibodies recognizing these proteins in zebrafish are not available. All markers were assessed in the livers of 5-dpf larvae exposed to the maximal tolerable dose of each drug from 3–5 dpf. All UPR sensors were significantly induced in the livers of Tm-treated larvae, whereas only some were induced in response to Tg and BFA (Fig. 2A–D). Thus, different stressors have distinct effects on each UPR sensor.

**Fig. 3.**
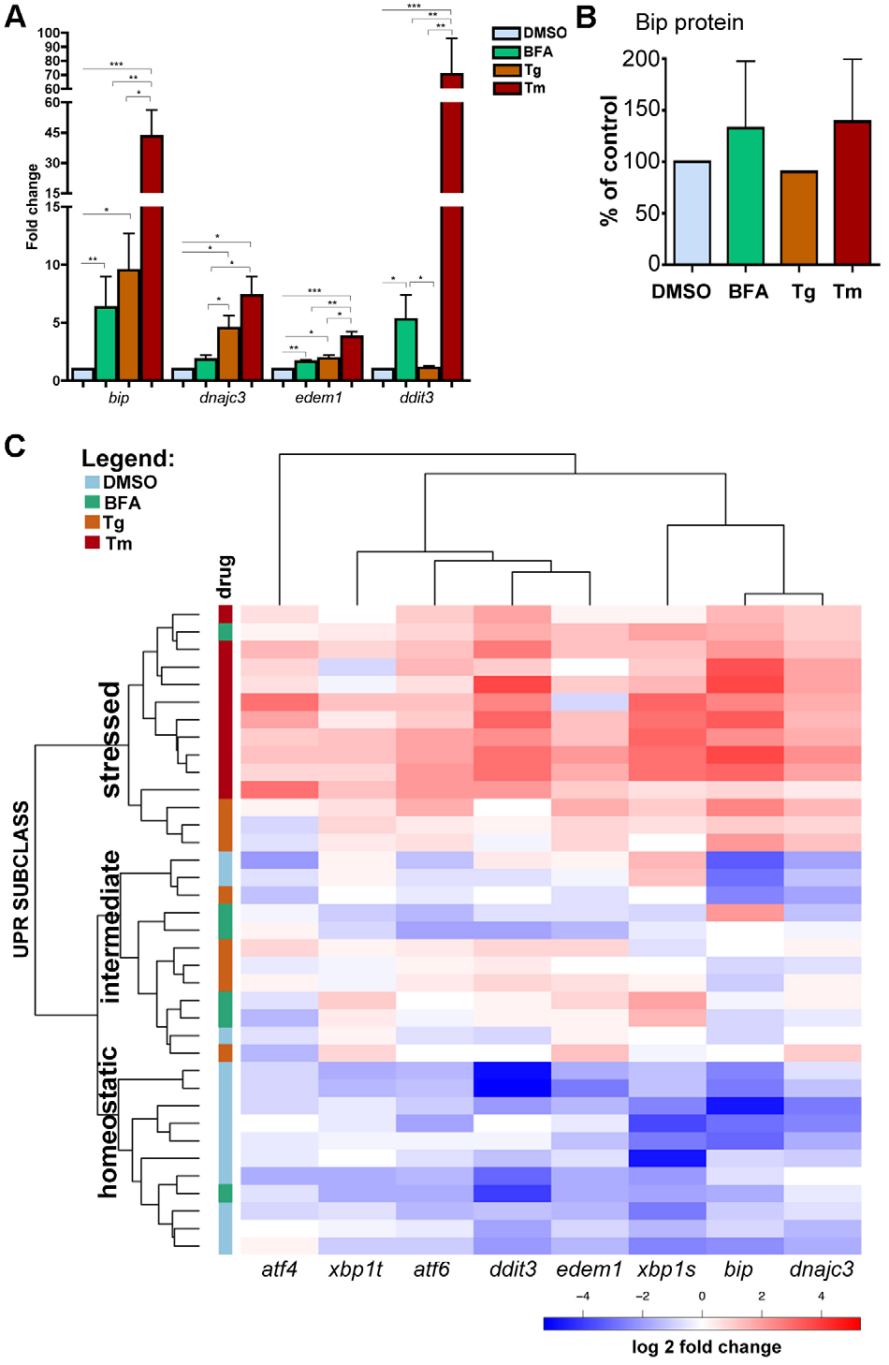
**BFA, Tg and Tm differentially induce UPR target genes in the liver.** (A) qPCR analysis of *bip*, *dnajc3*, *edem1* and *ddit3* in cDNA from dissected livers of 5-dpf larvae treated with DMSO, 1 μg/ml BFA, 0.75 μM Tg and 1 μg/ml Tm. The fold change relative to DMSO is plotted with error bars indicating the standard error; *n*=13 for DMSO, 10 for Tm, 8 for Tg and 6 for BFA. (B) Liver protein lysates from larvae treated as in A were immunoblotted with anti-*Bip* and anti-*tubulin* as a loading control. Band intensities were assessed, normalized to the loading control and plotted with error bars indicating the standard error; *n*=2. (C) Heat map of UPR target gene expression in the livers of larvae treated as in A with genes in columns and individual clutches of fish in rows. Red correlates with +Log 2 fold changes and blue correlates with −Log 2 fold changes, which are normalized across each column. Unsupervised clustering revealed three major UPR subclasses termed homeostatic, intermediate and stressed. Brackets reflect the results of unsupervised clustering of the genes and samples. *P<0.05, **P<0.01, ***P<0.001, calculated using paired Wilcoxon test.

qPCR was used to compare the expression of four UPR target genes, the chaperones *bip* and *dnajc3*, *edem1* and *ddit3* (also called *chop*; Fig. 3A). Immunoblotting was used to measure Bip protein in the liver of larvae exposed to each stressor (Fig. 3B; supplementary material Fig. S2B). All markers were significantly and highly upregulated in Tm-treated livers, whereas only some were significantly induced in the other samples: *dnajc3* was upregulated nearly to the same extent by both Tm and Tg, but not significantly upregulated by BFA, whereas *ddit3* was upregulated by 5.3-fold and 78.7-fold in BFA- and Tm-treated livers, respectively, but was not induced at all by Tg (Fig. 3A). This also shows that the different effects are most clearly demonstrated from analysis of gene expression at the mRNA level, as the differences in Eif2a phosphorylation (Fig. 2C; supplementary material Fig. S2A) or Bip protein (Fig. 3B; supplementary material Fig. S2B) were not as dramatically different between stressors.

We thus expanded the qPCR analysis to include eight markers of UPR induction across multiple batches of fish treated with each stressor and controls. The unsupervised clustering of this expression data shown in the heat map in Fig. 3C reveals three distinct groups (i.e. subclasses) of gene expression patterns. The first group is predominated by Tm samples (red) and displayed high upregulation of all UPR target genes. A related subcluster contained most of the Tg samples (orange), which displayed high upregulation of all genes, except *atf4*. Together, we designate this subclass as *stressed* UPR. A second subclass was dominated by DMSO-treated samples (light blue), where all genes were expressed at the lowest levels. We designate this as *homeostatic* (or baseline) UPR. A co-clustering branch was populated by samples from all categories, including most BFA samples (green), and showed a heterogeneous expression profile where some genes were highly expressed in most samples (i.e. *edem1* and *xbp1t*) and others were expressed at low levels (*bip*). We termed this an *intermediate* subclass. These findings argue against the concept that the UPR functions as a rheostat, whereby the entire pathway is dialed up and down in sync. Instead, it suggests that different types of secretory pathway stress elicit distinct UPR subclasses in the liver. This is similar to the different ‘states’ that have been described using computational modeling of the UPR (Erguler et al., 2013).

### Tm-induced FLD is directly correlated with UPR activation

We hypothesized that the different UPR subclasses identified above are related to the differential ability of each stressor to induce steatosis. However, it is also possible that the distinct cellular defects induced by each stressor could dictate the outcome separately from their effects on the UPR. To differentiate between these possibilities, we used a range of concentrations of a single stressor (Tm) and assessed UPR activation and steatosis.

**Fig. 4.**
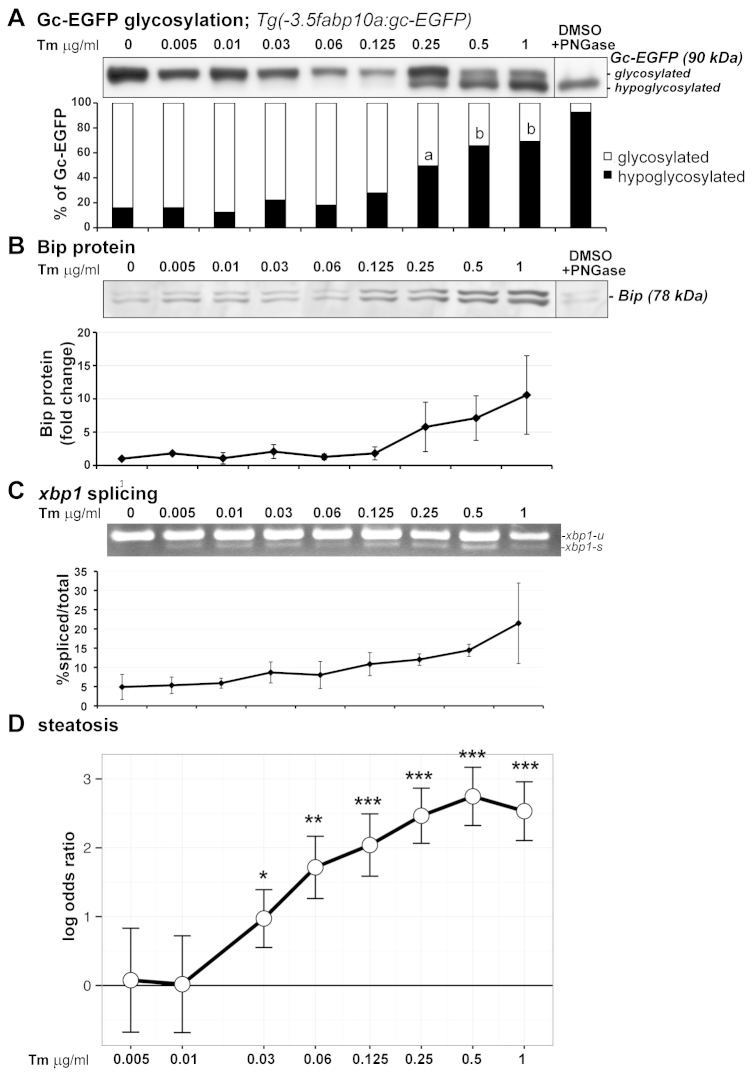
**Tm dose-response data reveals a nonlinear effect on hepatic protein glycosylation, Bip protein accumulation, *xbp1* splicing and steatosis incidence.** (A,B) Protein extracts from individual transgenic *Tg(l-fabp:Gc-EGFP)* larvae exposed to a range of Tm concentrations from 3 to 5 dpf were loaded in each lane and immunoblotted using anti-GFP (A) or anti-Bip (B). In A, PNGase treatment of extracts from DMSO-treated larvae revealed a faster migrating band corresponding to hypoglycosylated Gc-EGFP. Band intensities were plotted as the percentage of total Gc-EGFP that was either hypoglycosylated or fully glycosylated (*n*=3). One-way ANOVA was used to determine significance (*P*<0.05), where all samples marked with a letter are significantly different from DMSO controls, and those with different letters are significantly different from each other. In B, Bip protein levels in Tm-treated samples were compared with DMSO control and expressed as a fold change (*n*=2). (C) PCR analysis of unspliced (*xbp1-u*) and spliced (*xbp1-s*) *xbp1* mRNA in livers of 5-dpf larvae treated as in A. The percentage of *xbp1-second* from total *xbp1* is plotted (*n*=2). (D) Zebrafish larvae were treated as in A, stained with Oil Red O and scored for steatosis. Estimated log odds ratios of percentage steatosis between different concentrations of Tm and DMSO controls are plotted. *P<0.05, **P<0.01, ***P<0.005, calculated using paired Wilcoxon test.

**Fig. 5.**
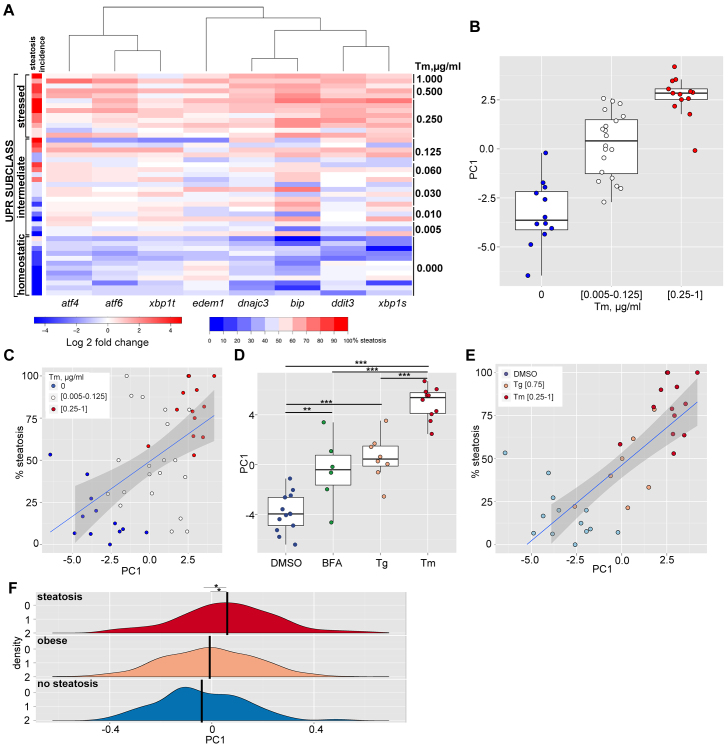
**UPR activation positively correlates with steatosis incidence.** (A) Heat map of UPR target gene expression in the liver, with genes in columns and individual clutches in rows. Blue and red indicates below and above average, respectively, for each column. Supervised clustering was performed according to Tm concentration (first) and steatosis incidence (second) and was unsupervised for gene order. Brackets reflect the results of unsupervised clustering of the genes. The colored bar on the left represents steatosis incidence (see legend: dark blue, 0%; white, 50%; dark red, 100%). The raw data are listed in supplementary material Table S2. (B) First principal component (PC1) of the UPR gene expression for each clutch was plotted according to Tm concentration, with the box indicating the 75th and 25th quartile of the data, the whiskers indicating the 90th and 10th percentile and the cross line indicating the median. (C) Scatter plot representing correlation between PC1 and steatosis incidence in larvae treated with different doses of Tm. Each dot represents a single clutch and is assigned a different color corresponding to UPR subclass (blue, white and red characterize homeostatic, intermediate and stressed UPR, respectively). A least squares linear model fit (blue line) with 95% prediction error bands (gray overlay) was superimposed. (D) Plot of PC1 for DMSO (control; blue), 1 μg/ml BFA (green), 0.75 μM Tg (orange) and 1 μg/ml Tm (red). (E) Scatter plot representing correlation between PC1 and steatosis incidence in larvae treated with DMSO (blue), 0.75 μM Tg (orange) and 0.25–1 μg/ml Tm (red) using the same model as in C. The correlation between PC1 and steatosis has *P*<0.001, determined by the Spearman test. (F) Kernel density estimate of the distribution of the UPR-PC1 signature in three sets of clinical samples. Per-group average is marked with a thick black vertical line. *P<0.1, **P<0.01, ***P<0.005, calculated using paired Wilcoxon test.

We first determined whether there was a correlation between a Tm-induced block in protein glycosylation, UPR induction and steatosis. To do this, we exposed 3-dpf larvae to sublethal concentrations of Tm (0.005–1 μg/ml; supplementary material Fig. S1E) or DMSO (control) for 48 hours. Tm efficacy was measured by assessing Gc-EGFP glycosylation (Fig. 4A). UPR induction was assessed by immunoblotting for Bip protein (Fig. 4B) from whole larval extracts, and by quantifying *xbp1* splicing (Fig. 4C) and UPR target gene expression (Fig. 5) in the liver. Immunoblotting extracts of whole larvae showed that hypoglycosylated Gc-EGFP increased from ~15% in untreated larvae to 50% in larvae treated with 0.125 μg/ml Tm and to almost 75% in larvae treated with 0.5 or 1 μg/ml Tm (Fig. 4A). We then blotted for Bip protein in these same larvae and found it to be elevated by exposure to Tm concentrations of 0.125 μg/ml or higher (Fig. 4B). *xbp1* splicing was increased considerably in the liver of larvae treated with Tm concentrations exceeding 0.125 μg/ml (Fig. 4C). Collectively, these data indicate that 0.125 and 0.25 μg/ml Tm are the lowest and maximal effective concentrations, respectively, that induce the UPR.

We next determined whether these markers of Tm efficacy correlated with steatosis incidence. We observe a range of 0–53% steatosis incidence across a large population of 5-dpf animals from two commonly used lines of fish (77 clutches with 1519 larvae), with a median of 10% (supplementary material Fig. S3A,B). Incidence increased as larvae aged, due to fasting-induced steatosis (supplementary material Fig. S3A). Thus, to determine whether Tm increased steatosis in a dose-dependent fashion, 15 clutches of control larvae and 4–10 clutches treated with eight concentrations of Tm were scored for the presence or absence of steatosis based on Oil Red O staining. A clutch-specific random effect term in a mixed effects logistic regression was used to determine whether Tm increased steatosis incidence. The log odds ratio between DMSO-and Tm-treated larvae from each clutch (*n*=1001 embryos from 4–15 clutches with a median clutch size of 14; Fig. 4D) revealed a significant increase in steatosis in larvae treated with 0.03 μg/ml (Fig. 4D; supplementary material Fig. S3B). These results were further supported by analysis with the nonparametric, paired Wilcoxon signed-rank test, which showed a significant increase in steatosis incidence at 0.03 μg/ml (two-sided *P* value of 0.04; Fig. 4D; supplementary material Table S1). Triglyceride concentrations measured in pools of livers dissected from 5-dpf larvae exposed to DMSO or 1 μg/ml Tm showed a range of values in each cohort, but the median hepatic triglyceride concentration in Tm-treated larvae was more than double that of controls (supplementary material Fig. S3C). We conclude that steatosis incidence is a highly sensitive assay for Tm efficacy *in vivo*.

### Gene expression analysis defines the relationship between a stressed UPR and steatosis

We tested whether the UPR subclass model that describes the relationship between gene expression and outcome was valid in response to the broad range of stress induced by varying concentrations of Tm. The panel of UPR genes was assessed by qPCR in the livers of a subset of the larvae treated with 0.005–1 μg/ml Tm from 3–5 dpf that were analyzed for steatosis incidence (Fig. 4D; supplementary material Table S2). Unsupervised clustering of gene expression profiles coupled with supervised clustering of clutches according to Tm concentration revealed three UPR subclasses (as shown in Fig. 3): a homeostatic UPR in livers of control larvae; an intermediate UPR characterized by variable induction of all genes in larvae exposed to 0.005–0.125 μg/ml Tm; and a stressed UPR characterized by high expression of all UPR genes in fish treated with 0.25 μg/ml Tm and higher (Fig. 5A). Importantly, all clutches with a stressed and intermediate UPR had a significantly higher steatosis incidence than the steatosis in all clutches in the homeostatic subclass (*P*<0.0001 and *P*=0.0014 by Wilcoxon test, respectively).

These data showed a clear correlation between high steatosis and a stressed UPR and low steatosis and a homeostatic UPR; however, samples in the intermediate subclass had variable increase in the expression of each gene. To clarify whether there was an increase in UPR gene expression across this broad continuum of stress, we used principal component (PC) analysis of the gene expression data in Fig. 5A, which allowed direct comparison of different experimental conditions in terms of the overall UPR level, instead of relying on individual analysis of each parameter. We identified the first principal component (PC1) that captured 72.5% of the total data variability and was thus a good synthesis of the data (supplementary material Table S3). Importantly, PC1 directly correlated with the doses of Tm that differentiated the three UPR subclasses (Fig. 5B). Correlation with steatosis showed that despite significant departures from a linear model fit, there was a distinct trend linking PC1 and steatosis incidence (Fig. 5C; supplementary material Table S4). Analysis of PC1 across the entire range of exposures demonstrated that PC1 increased even at the lowest Tm concentrations (supplementary material Fig. S4; Table S4). This also showed that there was the greatest variability in PC1 in the intermediate subclass, and that, in this subclass, the correlation between PC1 and steatosis incidence was the least robust, suggesting that additional parameters are required to more clearly define and increase significance of correlation with outcome. Regardless, these data support the conclusion that a median PC1 greater than 2.0 defines a stressed UPR and a median PC1 of less than −3.0 defines a homeostatic UPR (Fig. 5B; supplementary material Table S4).

We then used PC1 to test the hypothesis that BFA failed to induce steatosis because it did not induce a stressed UPR. We found that although PC1 was higher in both BFA- and Tg-treated samples compared with controls, it was substantially lower than that derived from the Tm-treated samples (Fig. 5D). Combined analysis of the Tg and Tm samples revealed a direct and linear correlation between PC1 and steatosis incidence that was independent of the stressor (Fig. 5E). These data indicate that full activation of UPR target genes, represented by PC1, is directly and linearly correlated with steatosis incidence. Finally, we demonstrated that steatosis can occur in the absence of UPR upregulation, as 6- and 7-dpf larvae that develop fasting-induced steatosis (supplementary material Fig. S3A) did not display any induction of UPR target genes in the liver (supplementary material Fig. S5).

### UPR target gene expression is upregulated in human FLD samples

We next determined the predictive power of our findings from zebrafish to human datasets. We mined previously published microarray data from liver samples from a series of 427 Caucasian patients with steatosis of unknown etiology, from those in the same cohort without documented steatosis (Schadt et al., 2008) and from a series of nearly 1000 morbidly obese (BMI >50) patients (Greenawalt et al., 2011), the majority of whom were predicted to have steatosis. We assessed the expression of *ATF4*, *ATF6*, *BIP*, *DDIT3*, *DNAJC3* and *EDEM1*, but *XBP1S* and *XBP1T* were not measured by the microarray, and thus could not be included (supplementary material Table S5). We observed a significant overexpression of *DNAJC3*, *EDEM1* (*q* value <0.01) and *BIP* (*q* value = 0.055) in patients with severe steatosis, and of *DNAJC3* (*q* value = 0.023) and *EDEM1* (*q* value = 0.014) in obese patients, compared with patients with no documented steatosis (supplementary material Table S5).

The different methods of gene expression analysis (i.e. qPCR in zebrafish and microarray in humans) prohibited direct comparison of absolute expression levels between zebrafish and human samples and we did not have access to the tissue used in these studies to perform direct measurements. Due to these constraints, we could not use the cut-off of median PC1 exceeding 2 to determine whether these samples had a stressed UPR. Regardless, we concluded that despite the overlap of PC1 across cohorts, there was a significant increase in PC1 in livers from patients with documented steatosis (Schadt et al., 2008) and in samples from morbidly obese patients (Greenawalt et al., 2011) compared with controls (*P*<0.01; Fig. 5F).

### UPR activation in response to Tm precedes steatosis

**Fig. 6.**
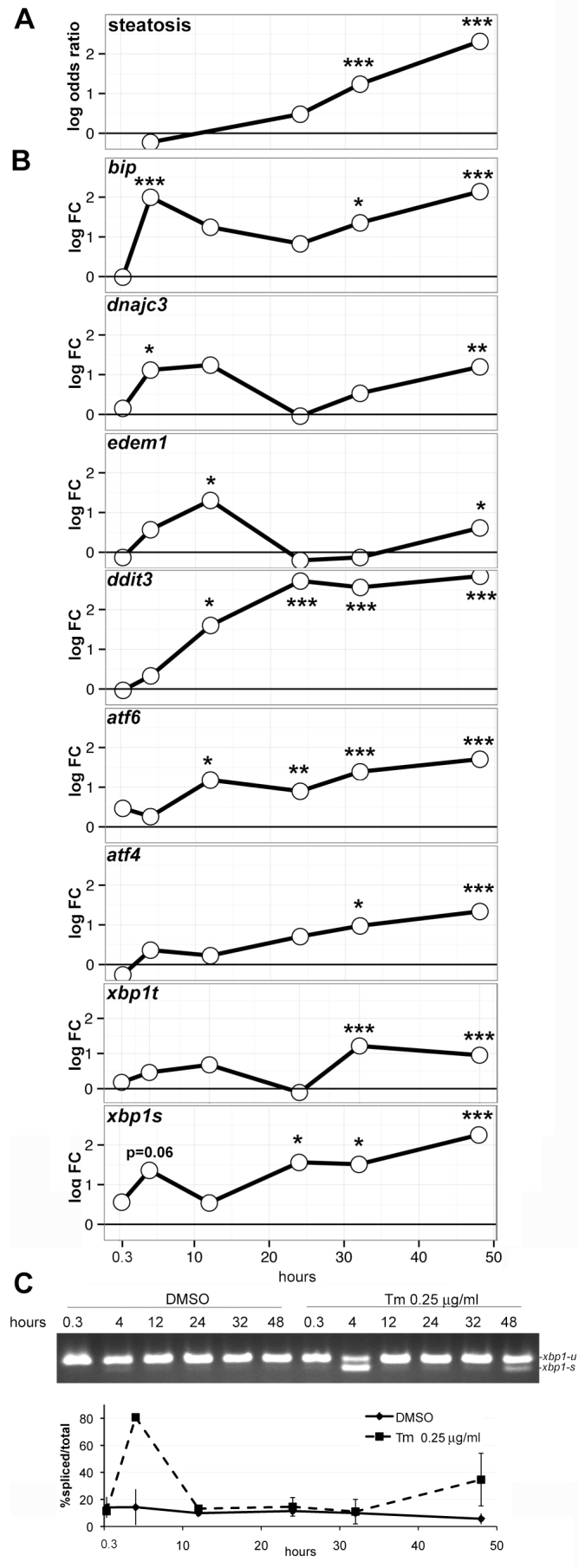
**UPR target gene activation precedes Tm-induced steatosis.** (A–C) Larvae were treated starting on 3 dpf with DMSO and 0.25 μg/ml Tm and collected at the indicated time points. At least 15 larvae were stained with Oil Red O and scored for steatosis (A) and at least 5 livers were dissected for RNA extraction (B,C). (A) The log odds ratio of steatosis was plotted versus time (see Materials and Methods and supplementary material Table S3). (B) UPR target gene analysis measured by qPCR in liver cDNAs from the same cohorts assessed for steatosis in A. Expression of each gene is plotted as log fold change (log FC) over time (see Materials and Methods and supplementary material Table S7). (C) PCR analysis of *xbp1* splicing with the percentage of spliced/total *xbp1* for each sample plotted (*n*=2). *P<0.05, **P<0.01, ***P<0.001.

We next assessed which aspects of the UPR were induced prior to steatosis by scoring for steatosis incidence and for UPR activation in larvae treated with 0.25 μg/ml Tm starting on 3 dpf for 0.3, 4, 12, 24, 32 and 48 hours (Fig. 6; supplementary material Table S6). Steatosis incidence was 63% and 80% at 32 and 48 hours of Tm exposure, respectively, the only time points that showed a significant increase compared with controls (*P*<0.001; Fig. 6A; supplementary material Table S7). As early as 20 minutes (0.3 hours) of Tm exposure resulted in a modest, but not significant, induction of *atf6* expression and *xbp1* splicing (Fig. 6B,C). After 4 hours of exposure, *bip* and *dnajc3* were significantly induced. However, the expression pattern of all genes was highly dynamic. Most genes displayed a biphasic induction pattern, whereby they were induced early (between 4 and 12 hours), were then downregulated (between 12 and 24 hours), reactivated again by 32 hours and remained fully induced by 48 hours of exposure (Fig. 6B; supplementary material Table S6). This dynamic pattern resulted in a low degree of coexpression of these genes (Fig. 6B; supplementary material Table S7), which made PC1 difficult to interpret and less appropriate for these series. Collectively, our findings further support the subclass model whereby unique UPRs are activated at different times during the stress response.

### An adaptive UPR reduces Tm-induced steatosis

Our *in vivo* data reflect other studies using cells in culture where acute response to Tm is characterized by robust activation of all UPR target genes and, if this is sufficient to manage the stress, the UPR is downregulated. Interestingly, cells that can overcome stress are better able to withstand exposure to subsequent stress, and are thus considered adapted (Rutkowski and Kaufman, 2007).

**Fig. 7.**
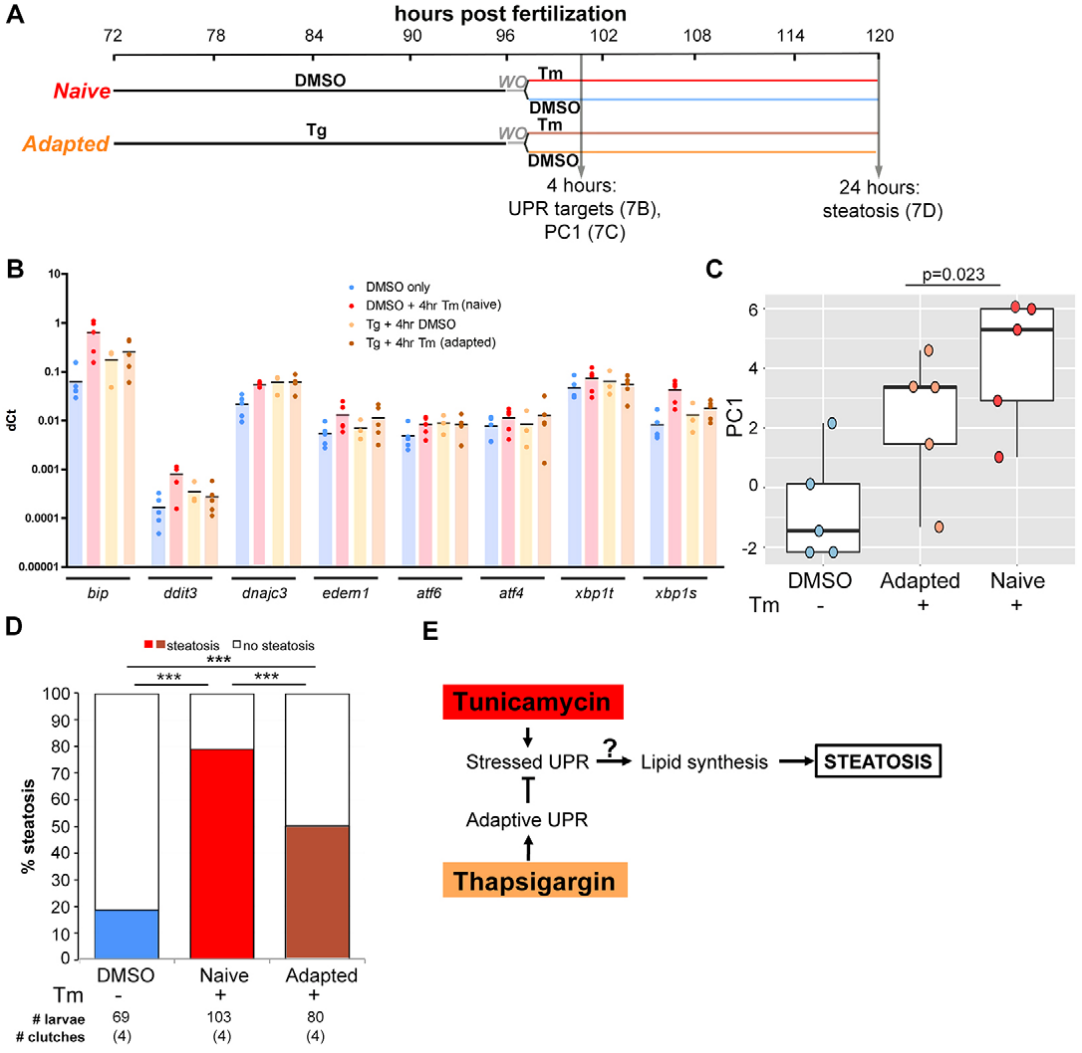
**An adaptive UPR protects against Tm-induced FLD.** (A) Experimental design for the adaptation protocol. Larvae were treated starting at 72 hpf with 0.75 μM Tg (adapted) or DMSO (naïve) for 24 hours, and the compounds were then washed out (WO) for 1 hour and the larvae were challenged with exposure to 0.25 μg/ml Tm or DMSO (control). Livers were dissected after 4 hours of exposure for qPCR analysis (B), for PC1 analysis (C) and after 24 hours of exposure fish were stained with Oil Red O (D). (B) ΔCt values of UPR genes in the livers of larvae treated as in A and collected at 101 hpf, after 4 hours of Tm challenge. Black lines indicate the median. (C) PC1 generated from qPCR analysis in B from samples treated as in A were plotted against the different treatments. See also supplementary material Table S8. (D) Larvae were treated with DMSO or Tg and then challenged with Tm and collected at 5 dpf as shown in A, stained with Oil Red O and scored for steatosis. (E) Model depicting the mechanism whereby Tg pretreatment induces an adaptive UPR that protects against Tm induction of a stressed UPR and steatosis. ***P<0.005, calculated using Fisher’s exact test.

We hypothesized that an intermediate UPR might reflect an adaptive UPR that could reduce the induction of a stressed UPR and steatosis by Tm. To test this, we developed an adaptation protocol as shown in Fig. 7A: larvae were treated with either DMSO (naïve) or Tg (adapted) for 24 hours starting on 3 dpf to generate an adaptive UPR and, following an hour of recovery time, they were exposed to 0.25 μg/ml Tm for 24 hours to generate a stressed UPR that was comparable to that detected in fish treated for 48 hours (supplementary material Fig. S6). Adaptation is demonstrated by a reduction in UPR target gene expression following a robust stressor. Most of the UPR target genes that were induced by 4 hours of Tm exposure in naïve fish were reduced in adapted fish (Fig. 7B) and this was clearly demonstrated by the significant reduction in PC1 in the liver of adapted larvae (Fig. 7C; supplementary material Fig. S7). Although this reduction was mild (log fold change, −1.9585; *t* value, −2.3597; one-sided *P* value, 0.023), within the signature, *bip*, *ddit3* and spliced *xbp1* showed a strongly decreased level of expression (log fold change, −1.2727, −1.4660 and −1.2107; one-sided *P* values, 0.0238, 0.0041 and 0.0012, respectively; see supplementary material Table S8).

We found no increase in steatosis after the 24 hours of Tg treatment compared with DMSO-treated controls (not shown). Importantly, although nearly 80% of naïve larvae developed steatosis after the Tm challenge, this was reduced by almost half in adapted larvae (Fig. 7D). These results are consistent with the hypothesis that adaptation to secretory pathway stress reduces the induction of a stressed UPR and therefore reduces steatosis, which is similar to findings in mammals (Achard and Laybutt, 2012; Ye et al., 2010).

The mechanism by which Tm induces steatosis is not known. One study in mice (Lee et al., 2012) suggested that Tm induces hepatic lipid synthesis by activating the sterol response element binding proteins (Srebps). However, we found no evidence of Srebp activation in the liver of Tm-exposed fish as measured by expression of target genes (supplementary material Fig. S8A). Similarly, Tm did not alter expression of genes that regulate the hepatic insulin response (supplementary material Fig. S8B). This suggests that, unlike our findings in alcoholic FLD (Passeri et al., 2009; Tsedensodnom et al., 2013), Srebp-mediated lipogenesis is not a major contributor to steatosis caused by a stressed UPR. This is consistent with our recent finding that active Atf6 is sufficient to induce steatosis via an Srebp-independent mechanism (Howarth et al., 2014).

## DISCUSSION

This study provides a novel perspective on the well-established link between UPR activation and FLD. Many studies have found some aspects of the UPR induced in FLD samples, and this has led to an oversimplification of the relationship between UPR activation and FLD, whereby any indicator of UPR activation is equated with ER stress and that all stressors that generate UPR induction cause FLD. Our data challenge this dogma by showing that (i) the UPR does not function as a rheostat in which all components behave synchronously in response to stress, but instead is characterized by distinct subclasses that change based on the nature and duration of the stress and (ii) some stressors induce a UPR subclass that causes FLD whereas other UPRs do not. Furthermore, our data confirm findings in other systems that an adaptive UPR can protect against the negative consequences of a robust stressor (Achard and Laybutt, 2012; Cinaroglu et al., 2011; Lugea et al., 2011; Rutkowski et al., 2006; Ye et al., 2010) and identify three subclasses that had been confirmed by modeling studies (Erguler et al., 2013).

Yeast and mammalian cell cultures have traditionally been the systems of choice for studying the UPR and little is known about UPR regulation and outcomes in whole animals. We used zebrafish larvae to address the differential consequences of a range of stressors using fatty liver as a clinically relevant outcome, and confirmed the relevance of these findings to humans with FLD. Zebrafish provide an advantage for such studies because it is possible to analyze a large range of markers of secretory pathway function and UPR activation across a large population – for instance, more than 5000 animals were scored for steatosis in this study. Furthermore, using computational methods to devise a relationship between a UPR target activation profile and steatosis, a detailed and nuanced portrait of the *in vivo* response of the liver to different stressors was generated. This provides an important first step towards understanding the hepatocyte stress response and a framework for identifying the players in the UPR that influence metabolic pathways and drive FLD.

We draw three main conclusions from this study. First, UPR activation causes steatosis, not vice versa, as fasting-induced steatosis is not accompanied by induction of UPR target genes, which is similar to findings in other systems (Congiu et al., 2011; Ren et al., 2012). Although these data also show that there are other pathophysiological mechanisms of FLD that do not involve UPR activation, our recent report that overexpression of active Atf6 in hepatocytes is sufficient to cause steatosis in zebrafish larvae (Howarth et al., 2014) demonstrates that this key UPR player is probably an important mediator of this disease in some instances. Occasionally, it is also possible that once steatosis occurs, oxidative damage to the ER generated by lipid peroxidation (Achard and Laybutt, 2012; Malhi and Gores, 2008) could exacerbate the stress and contribute to prolonged or enhanced UPR activation.

Second, our data support a ‘subclass’ model of UPR activation that dictates outcome. In this model, the outcome is determined by the distinct combinations of UPR sensors and effectors activated during the stress response. Similar findings in mammalian cells treated with different stressors and *in silico* modeling have shown that both the amplitude and kinetics of induction of UPR sensors and target genes show distinct patterns (DuRose et al., 2006; Erguler et al., 2013). Remarkably, even yeast, which possess only a single UPR branch orthologous to the vertebrate ERN1/XBP1 pathway, are capable of mounting unique UPR target gene profiles in response to different stressors (Thibault et al., 2011). Here, we have extended these studies using a whole vertebrate to demonstrate that unique UPR subclasses are activated by different stressors and that these change over time. This suggests that the UPR subclass activated as an early response to stress is transformed into a different subclass as the stress is either resolved (i.e. adaptive) or persists (i.e. stressed or terminal). This model can also be refined to include the concept of a threshold, whereby a homeostatic UPR allows cells to mitigate stress levels that are typically encountered under physiological conditions, but when the system becomes overwhelmed, the UPR acutely increases to execute different outcomes. A practical implication is that a single measure of UPR activation does not provide an accurate indication of pathway engagement. Even using a panel of eight parameters is insufficient to provide robust subclassification of the intermediate UPR and of the dynamic UPR that is activated during the time course of Tm-induced stress. Although a stressed UPR is most clearly identified using PC analysis of gene expression based on these parameters, expanding the range of parameters through whole transcriptome analysis and developing additional, quantitative measurements to assess activation of each UPR sensor would allow these UPRs to be refined.

Third, an adaptive UPR can protect against FLD. Our working model (shown in Fig. 7E) is that larvae exposed to a low level of stress do not fully activate a stressed UPR in response to a Tm challenge, although this effect is less pronounced at later time points (not shown). Importantly, adaptation reduces steatosis incidence, similar to the ability to protect against stress-induced apoptosis (Rutkowski et al., 2006), stress-induced pancreatitis (Lugea et al., 2011) and FLD (Achard and Laybutt, 2012; Ye et al., 2010). Thus, it is possible that promoting an adaptive UPR might be beneficial for treatment or prevention of diseases that are caused by high UPR induction.

We are encouraged that findings from zebrafish are predictive of phenotypes found in human FLD patients. However, there are several caveats to interpreting these data, and further analysis is warranted. First, although most morbidly obese patients have FLD (Merriman et al., 2006), this was not measured directly in the patients that were used for our analysis (Greenawalt et al., 2011), prohibiting a direct comparison. Moreover, the incomplete clinical information for most of the patients in these large cohorts, the different approaches used to assess gene expression in zebrafish and human samples, and the lack of *xbp1s* and *xbp1t* on the human microarray limit the robust analysis of these data. Nevertheless, despite all these caveats, our data provide a first indication that multiple parameter analysis of the UPR can capture the natural variability of the UPR in humans subjected to a variety of acute and chronic stresses.

The mechanism by which UPR activation causes lipid accumulation in hepatocytes is not known. Some studies suggest that UPR activation blocks insulin receptor signaling (Achard and Laybutt, 2012; Ozcan et al., 2004; Yang et al., 2010; Ye et al., 2010) to promote FLD, whereas others suggest that UPR sensors directly regulate the transcription of lipid metabolic genes (Lee et al., 2003; Rutkowski et al., 2008). We found that neither the Srebp pathway nor key players in insulin signaling change their expression in response to Tm exposure (supplementary material Fig. S8). This is consistent with our finding that Atf6 overexpression causes steatosis, in part due to induction of lipid synthesis by an Srebp-independent mechanism (Howarth et al., 2014). Indeed, our preliminary data demonstrate that Atf6 depletion protects against steatosis caused by 0.25 μg/ml Tm (not shown), suggesting that Atf6 induction in response to Tm might function by the same mechanism.

An intriguing alternative is that lipid accumulation in response to high levels of unfolded protein accumulation in the ER could serve a cell-protective effect, as clearing unfolded secretory cargo presents a significant demand for ATP. The lipid droplets might provide a metabolic advantage to allow hepatocytes to cope with the energetically demanding process of folding or degrading these proteins. As such, steatosis could be viewed as a means to recover from a stress instead of as an indication of liver disease.

## MATERIALS AND METHODS

### Zebrafish husbandry and transgenic lines

Wild-type (TAB14 or AB) lines of zebrafish were maintained in accordance with the policies of the Mount Sinai Institutional Animal Care and Use Committee. *Tg(fabp10:RFP)* and *Tg(−3.5fabp10a:gc-EGFP)^lri500^* [or *Tg(l-fabp:Gc-EGFP)*] fish were previously described (Korzh et al., 2008; Xie et al., 2010).

### Fasting

Larvae typically utilize their yolk by 5 dpf, after which they become fasted if they are not supplied with external nutrients. Larvae were maintained in Petri dishes at a concentration of no more than 1 larvae/ml and collected on 5, 6 and 7 dpf for analysis by Oil Red O staining or their livers were dissected to collect RNA for gene expression analysis.

### Treatments

Between 40 and 60 larvae were treated in 10-cm Petri dishes on 3 dpf with each stressor diluted in 40 ml of water. BFA (Calbiochem, EMD Chemicals), Tg (Santa Cruz) or Tm (Calbiochem, EMD Chemicals) were diluted in egg water (0.6 g/l Crystal Sea Marinemix; Marine Enterprises International) from a 10 mg/ml stock in DMSO. Control larvae were exposed to equal amounts of DMSO, which never exceeded 0.01%. For the adaptation experiments, larvae were pretreated at 3 dpf with 0.75 μM Tg, washed out for 1 hour and treated with 0.25 μg/ml Tm for 24 hours (adapted). In parallel, larvae were exposed to DMSO (control) or Tm for the same amount of time, without pretreatment (naïve).

### Triglyceride determination

Triglyceride measurements were carried out as described (Tsedensodnom et al., 2013). Briefly, 20 livers were dissected from 5-dpf larvae and pooled in 0.5% Triton X-100 (VWR International, West Chester, PA, USA) and the Infinity™ Triglyceride Liquid Stable Reagent (Thermo Fisher Scientific, Waltham, MA, USA) was used following the manufacturer’s instructions. Triglyceride levels were normalized to the total protein concentration as determined by BCA Assay (Thermo Fisher Scientific, Waltham, MA).

### Western blotting

Individual larva or pools of 15–20 livers dissected on 5 dpf were lysed and processed for immunoblotting as described (Cinaroglu et al., 2011). Protein concentration was determined by the BCA method (Thermo Fisher Scientific, Waltham, MA). Antibodies recognizing β-actin (A2228, Sigma), Bip (ET-21, Sigma), GFP (632569, Clonetech) and phosphorylated Eif2a (9721S, Cell Signaling), followed by the HRP-conjugated secondary antibody (anti-mouse or anti-rabbit; Promega), were visualized by chemiluminescence using the FujiFilm LAS-3000. Quantification of band intensities was performed using ImageJ software (http://rsbweb.nih.gov/ij/download.html).

Lysates of single, whole 5-dpf *Tg(l-fabp:Gc-EGFP)* larva were incubated with PNGase enzyme (New England BioLabs) for 1 hour at 37°C and processed for immunoblotting as described.

### PCR and qPCR

Pools of three to ten livers were dissected from 3- to 5-dpf larvae, homogenized and RNA extracted using TRIzol (Invitrogen). cDNA was obtained using qScript™ cDNA SuperMix (Quanta Biosciences). PCR to detect *xbp1* splicing was performed as described (Cinaroglu et al., 2011). Real time quantitative PCR (qPCR) was performed using the PerfeCTa SYBR Green FastMix kit (Quanta Biosciences) and the Roche LightCycler 480 System. Primer sequences are listed in supplementary material Table S9.

### Oil Red O staining

Whole mount Oil Red O staining was carried out as described (Cinaroglu et al., 2011). Larvae were scored as positive if more than five lipid droplets were observed in the hepatic parenchyma. Data on steatosis incidence by Tm dose was modeled using a mixed effects logistic regression, with fixed effects for the dose, coded as a categorical variable, and a constant random effect for the clutches, to account for clutch-to-clutch variations. The logistic mixed effect model was estimated using the lme4 R package (Bates et al., 2012).

### Dose response data clustering and principal component analysis

Clutches for which expression data was available for all eight genes were selected for analysis. The anti-Pearson correlation between log-intensities was used as a dissimilarity measure between genes. Principal component analysis was performed on the same data matrix using the ‘prcomp’ function in the ‘stats’ R package. Estimated first principal component (PC1) coefficients are reported in supplementary material Table S3.

The impact of Tg pretreatment on UPR gene expression and on PC1 (supplementary material Table S3) was evaluated using an ANOVA with three factors: treatment regime (control, naïve or adapted), clutch number (from 1 to 5) and experiment number (from 1 to 3). The comparison between naïve and adapted samples was tested with a one-sided contrast, testing for decrease in the signature in the adapted compared with naïve samples only. Gene expression was measured as log2-deltaCT (supplementary material Table S8).

### Human sample data analysis

Microarray gene expression data from 427 Caucasian patients (127 with steatosis) was obtained from Gene Expression Omnibus (GEO; http://www.ncbi.nlm.nih.gov/geo), super series accession number GSE9588, and was previously described (Schadt et al., 2008). Microarray gene expression data from 651 morbidly obese patients was obtained from GEO, super series accession number GSE24335, and was previously described (Greenawalt et al., 2011).

Differential expression of UPR genes in patients with different degrees of steatosis was assessed within the GSE9588 cohort with a linear model, including age and gender as covariates. Steatosis was reported in three levels: mild, moderate and severe, and these were pooled to a single level. *P*-values were adjusted for multiple testing across all the microarray probes using the Benjamini-Hochberg method.

Data from GSE24335 and GSE9588 were produced with the same microarray platform and were combined using ComBat (Johnson et al., 2007) to control for potential batch effects.

The PC1 of the zebrafish model Tm dose-response data was computed on the combined clinical data matrix by first matching genes to probes by gene symbol. PC1 genes that were not covered by the microarray were omitted. Multiple probes aligning to the same gene were averaged together.

### Kinetic analysis of gene expression

Time course steatosis and gene expression data were modeled with logistic (for the steatosis incidence) and linear (for the log2-gene expressions) mixed effects models, with fixed effects for the interaction between time point and treatment and a constant random effect for the clutches, to account for within-clutch correlations.

The mixed effects models were estimated using the lme4 R package (http://cran.r-project.org/web/packages/lme4/index.hTml) (Bates et al., 2012) and differences between treatments by each time point were tested using the ‘linearHypothesis’ function in the ‘car’ R package (Fox and Weisberg, 2011). Results of the comparisons between treatments by time point are reported in Fig. 6 and supplementary material Table S2. Multiple testing issues (eight genes by six time points, equals 48 tests) were mitigated by applying a Benjamini-Hochberg correction to *P*-values for gene expression comparisons.

## Supplementary Material

Supplementary Material
