# Supplementary material for: Molecularly defined unfolded protein response subclasses have distinct correlations with fatty liver disease in zebrafish
Source: Dis Model Mech. 2014 Jul;7(7):823–35. doi: 10.1242/dmm.014472 (PMC4073272; doi:10.1242/dmm.014472)
Supplement: Supplementary Material [file supp_7_7_823__index.html]

Molecularly defined unfolded protein response subclasses have distinct correlations with fatty liver disease in zebrafish — Supplementary Material 

# Molecularly defined unfolded protein response subclasses have distinct correlations with fatty liver disease in zebrafish

## DMM014472 Supplementary Material

**Files in this Data Supplement:**

- **Supplementary Material**
