# Supplementary material for: Molecularly defined unfolded protein response subclasses have distinct correlations with fatty liver disease in zebrafish
Source: Dis Model Mech. 2014 Jul;7(7):823–35. doi: 10.1242/dmm.014472 (PMC4073272; doi:10.1242/dmm.014472)
Supplement: Supplementary Material [file supp_7.7.823_DMM014472.pdf]

Figure S1

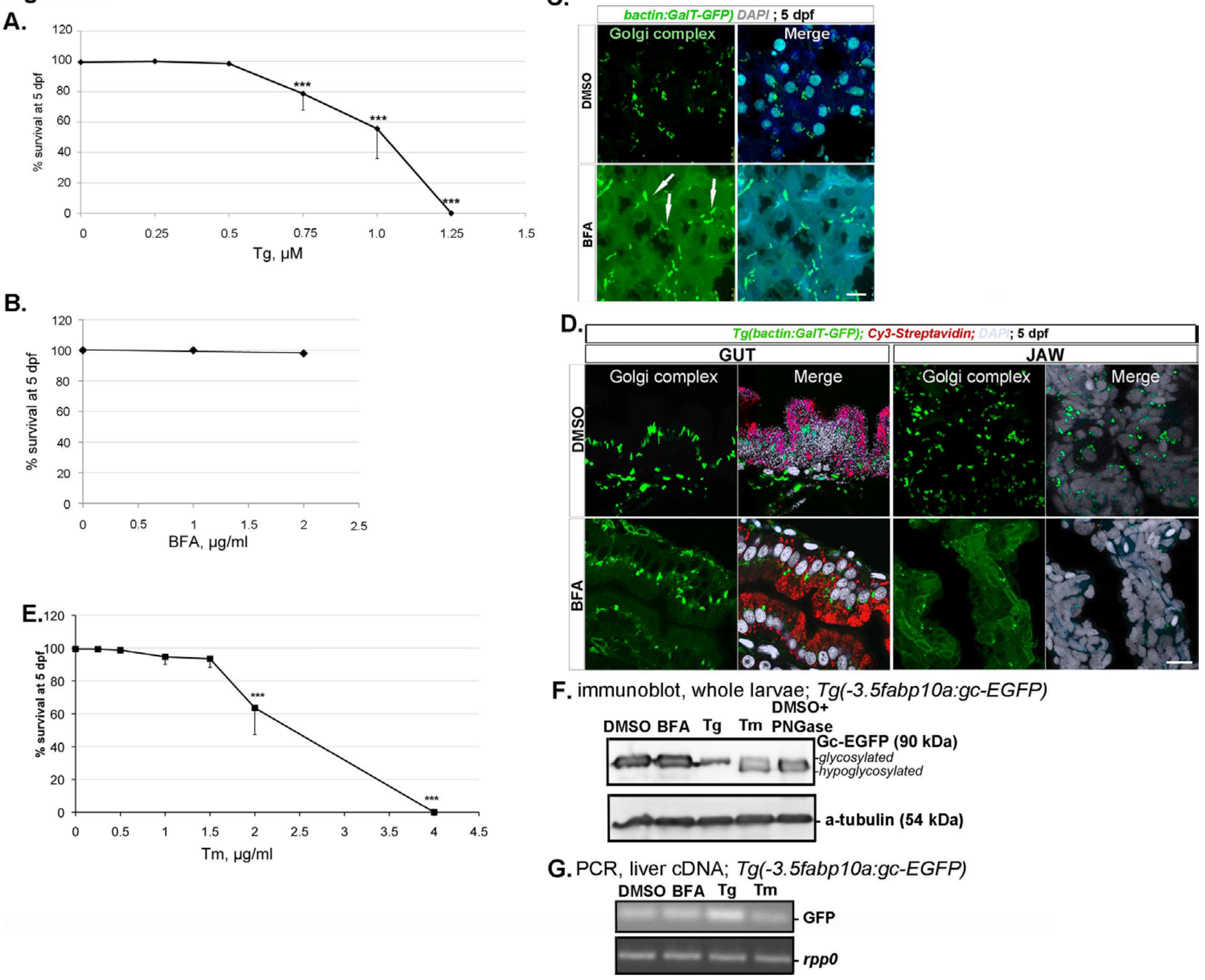

Fig. S1. Tg, BFA and Tm have different toxicities and affect differentially the secretory pathway morphology and function. A. Tg causes dose-dependent lethality of zebrafish larvae exposed from 3 to 5 dpf. **B.** Survival of zebrafish larvae treated with 1 and 2 µg/ml BFA from 3-5 dpf. **C.** Percent of larvae that survive exposure to Tm concentrations ranging from 0 to 4 µg/ml from 3 to 5 dpf was scored in at least 3 clutches. The maximal tolerable dose was determined as 1 µg/ml. **D.** Lysates of single 5 dpf *Tg(l-fabp:gc-EGFP)* larvae treated with DMSO, 1 µg/ml BFA, 0.75 µM Tg or 1 µg/ml Tm were immunoblotted with anti-GFP and anti-a-tubulin to detect a mobility shift in Gc-EGFP representing a change in glycosylation status. +PNGase indicates that protein lysates were treated with PNGase. **E.** PCR analysis of GFP and rpp0 from samples treated as in **D. F.** Confocal images of sections of hepatocytes from *Tg(bactin:GalT-GFP)* larvae treated with DMSO and 1 µg/ml BFA. Nuclei are stained with DAPI (blue). Scale bar = 10 µm. **F**. Confocal images of the gut and jaw from cryosections of transgenic 5 dpf *Tg(bactin:GalT-GFP)* zebrafish larvae treated from 3 to 5 dpf with DMSO (upper panels) or with 1 µg/ml BFA (lower panels) show a complete dispersal of the GC in case of the BFA treatment as compared with the DMSO control. Sections were stained with Cy3-streptavidin (red) to visualize the gut.

Figure S2

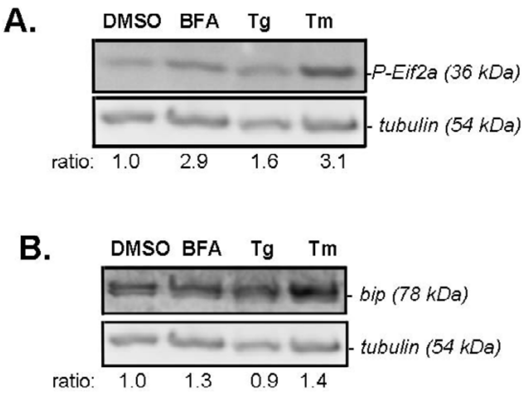

Fig. S2. BFA, Tg and Tm affect differentially expression of UPR targets. A-B. Western blot analysis of P-Eif2a and Bip in the livers of larvae treated with DMSO, 1 µg/ml BFA, 0.75 µM Tg and 1 µg/ml Tm from 3 to 5 dpf. Tubulin served as loading control. Band intensities were quantified and normalized to the DMSO controls.

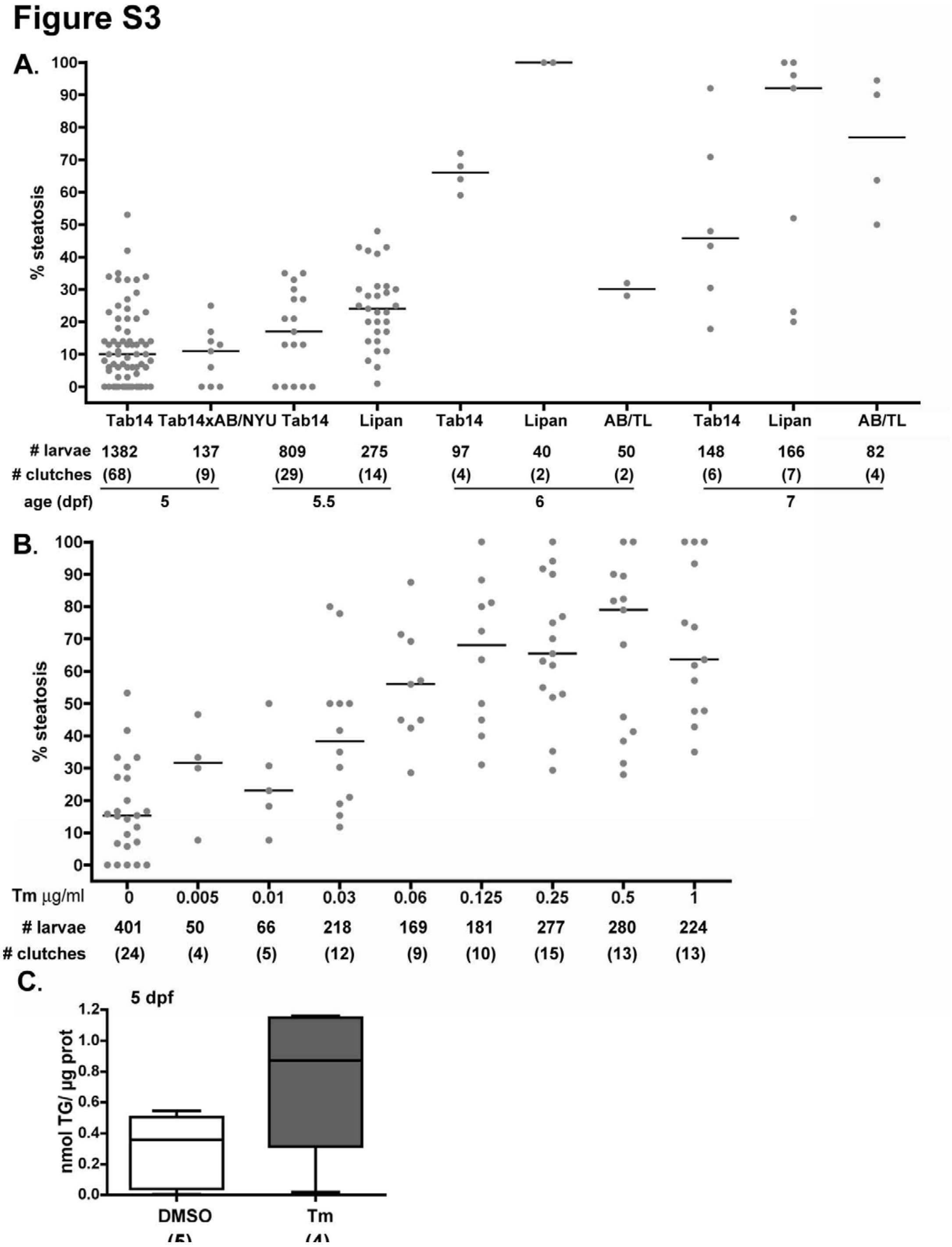

Fig. S3. Steatosis incidence in a large population of wild type zebrafish larvae at different ages. A. Larvae from different genetic backgrounds (Tab14, AB, LiPan=Tg(*fabp10:*RFP; *ela*:GFP), Tab14xAB) were collected on 5, 5.5, 6 and 7 dpf and stained with oil red O. The steatosis incidence for each clutch is plotted. Total number of scored fish is indicated, and the number of clutches is in parentheses. **B.** Steatosis incidence in a Tm dose response was quantified, with total number of larvae and clutches shown.. **C.** Total triglycerides were determined from livers of larvae exposed to 1 µg/ml Tm from 3 to 5 dpf and DMSO controls, and normalized to the total amount of protein

Figure S4

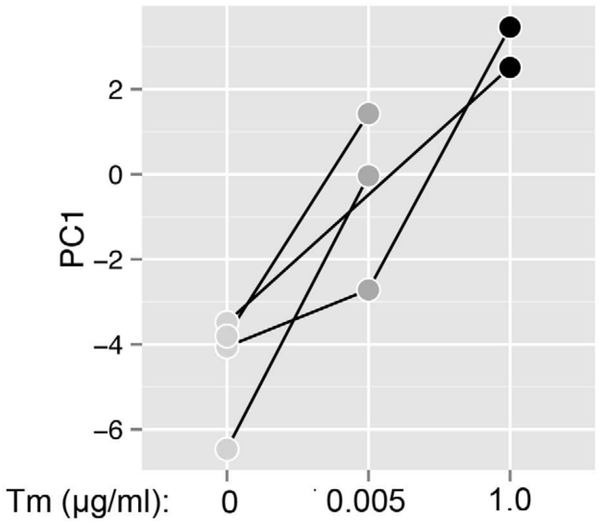

Fig. S4. 0.005 µg/ml Tm are sufficient to cause PC1 increase. Graph displaying UPR signature, PC1, plotted against the 0, 0.005 and 1 µg/ml Tm concentrations.

Figure S5

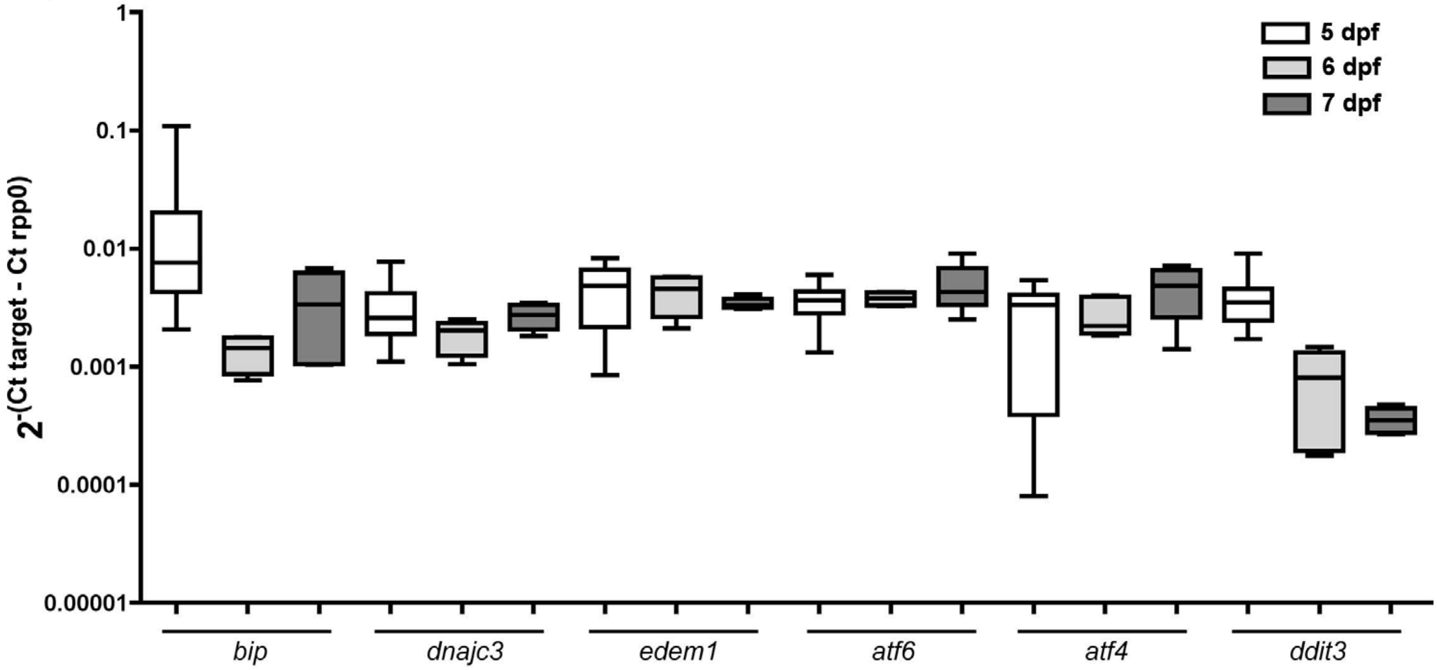

Fig. S5. Fasting does not induce a UPR response. qPCR analysis of UPR target genes and effectors in livers dissected from larvae at 5, 6 and 7 dpf.

Figure S6

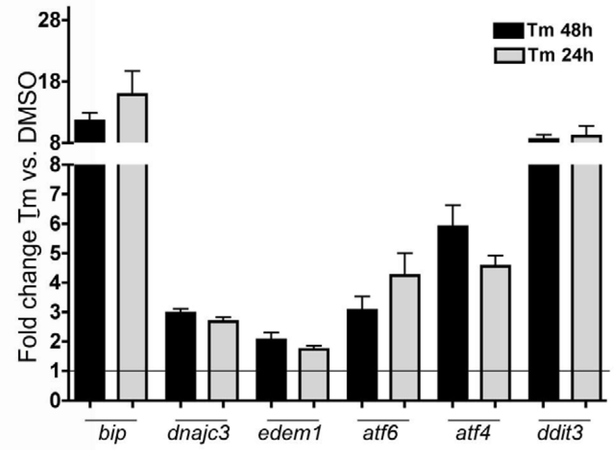

Fig. S6. Exposure to Tm for different time intervals results in similar upregulation of UPR target genes in the liver. Larvae were treated with 0.25 µg/ml Tm for the indicated time points and the expression of the UPR target genes was determined by qPCR.

Figure S7

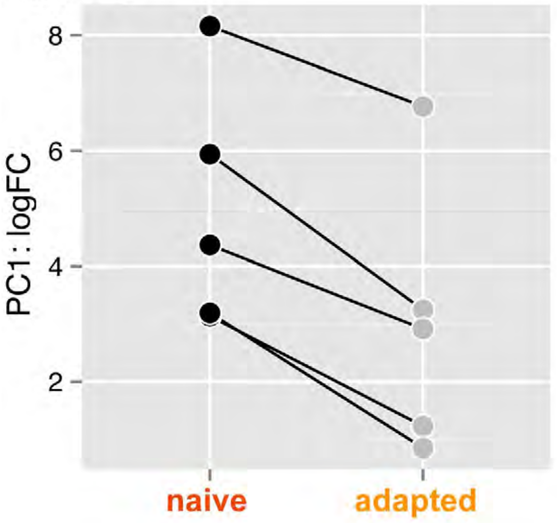

Fig. S7. Adaptation causes a decrease in PC1. Plot showing the PC1 in the naïve (DMSO, 24h, followed by Tm, 24h) and adapted (Tg, 24h, followed by Tm 24h) conditions. Note that in each experiment, the adapted PC1 is lower than the naïve PC1.

Figure S8

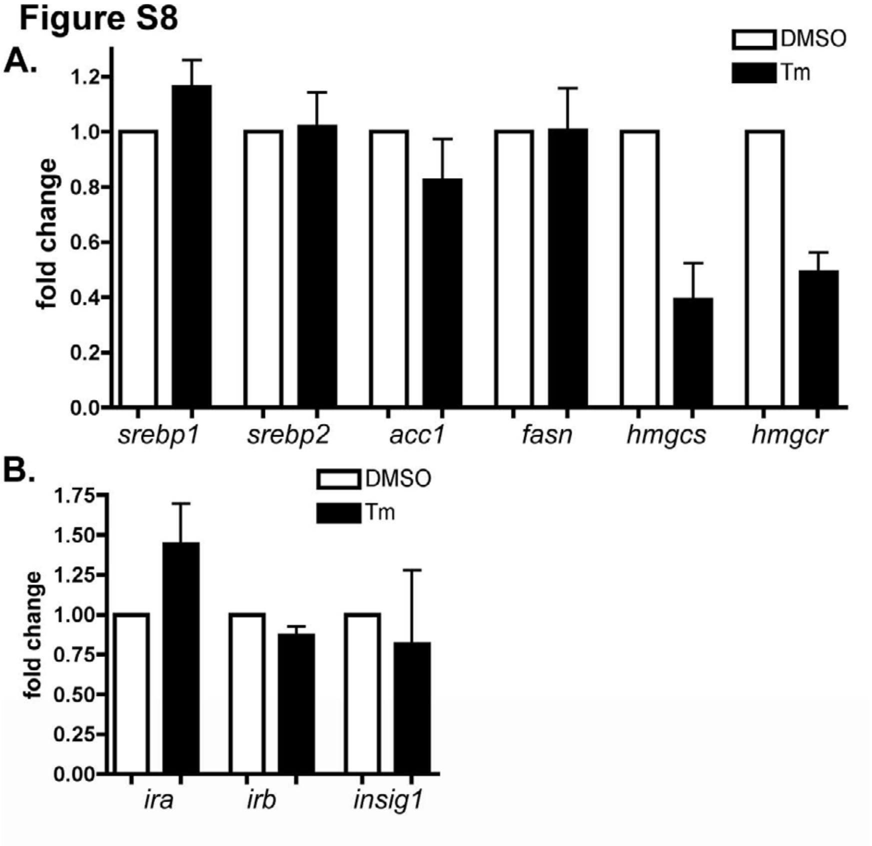

Fig. S8. 0.25 µg/ml Tm exposure does not cause induction of Srebps or insulin responsive genes. A. qPCR analysis of *srebp* response genes and their targets, *acc1, fasn, hmgcs* and *hmgcra* in the livers of 48h 0.25 µg/ml Tm treated larvae. Fold changes were expressed relative to the DMSO controls. **B**. qPCR analysis of insulin receptor *ira* and *irb* and also *insig1* in livers of larvae treated as in **A**.

Download Table S1

Download Table S2

Download Table S3

Download Table S4

Download Table S5

Download Table S6

Download Table S7

Download Table S8

Download Table S9
